# Supplementary material for: Novel Chemical Cross-Linked Ionogel Based on Acrylate Terminated Hyperbranched Polymer with Superior Ionic Conductivity for High Performance Lithium-Ion Batteries
Source: Polymers (Basel). 2019 Mar 7;11(3):444. doi: 10.3390/polym11030444 (PMC6473542; doi:10.3390/polym11030444)
Supplement: Supplementary file 1 [file polymers-11-00444-s001.pdf]

Electronic Supplementary Information

Novel chemical cross-linked ionogel based on acrylate terminated hyperbranched polymer with superior ionic conductivity for high performance lithium-ion batteries

Kang Zhao, Hongzan Song, Xiaoli Duan, Zihao Wang, Jiahang Liu, Xinwu Ba.

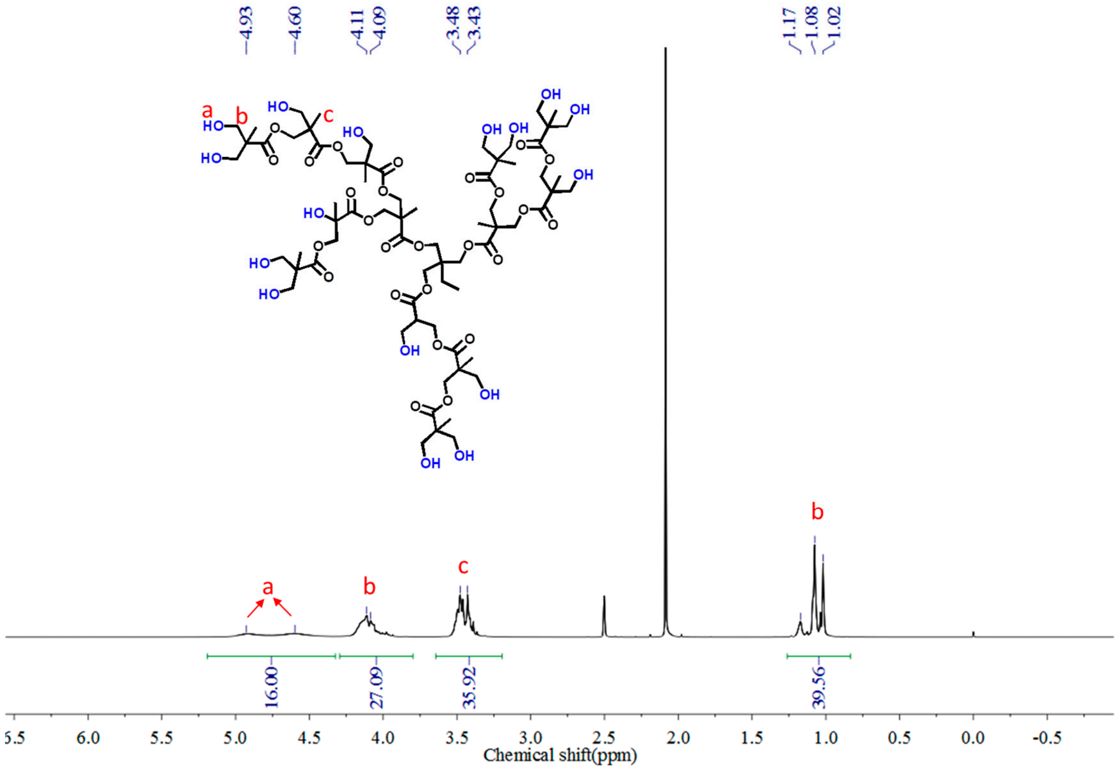

Figure S1 ^{1}H NMR spectra of HP-OH.

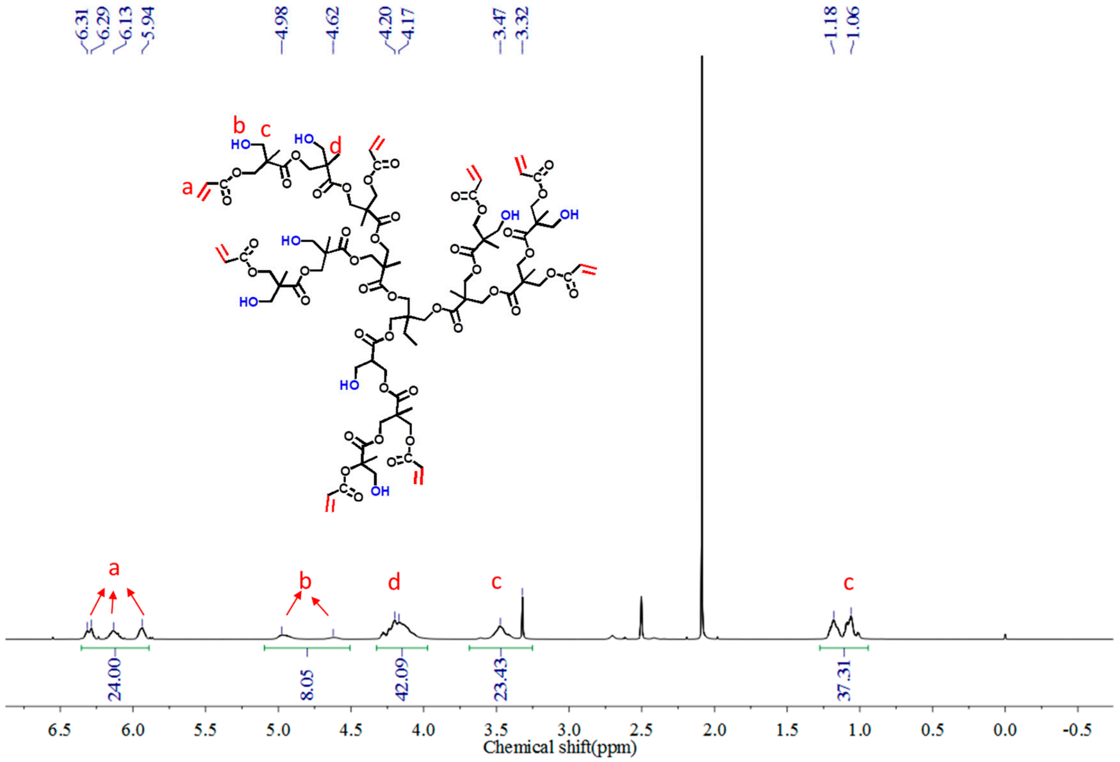

Figure S2 ^{1}H NMR spectra of HP-A.

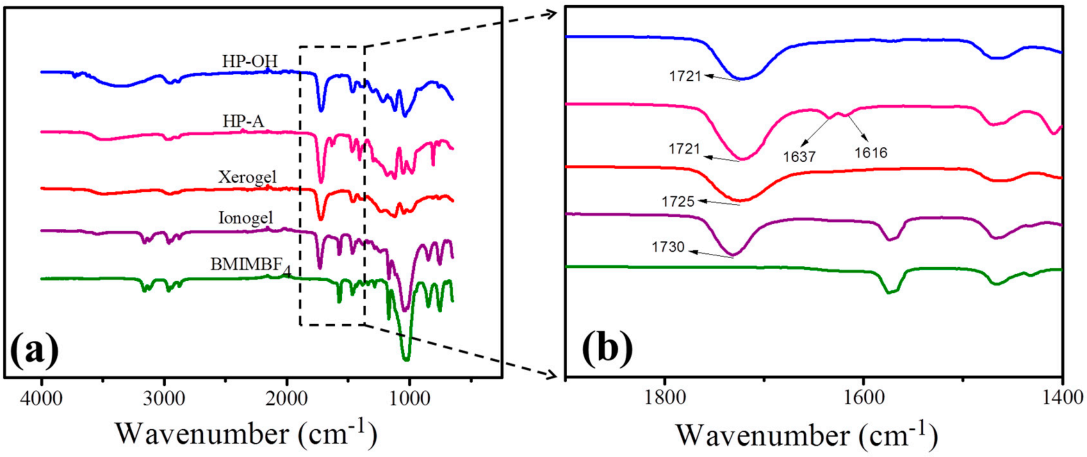

Figure S3 (a) FTIR patterns of neat HP-OH, HP-A, $BMIMBF_4$, Xerogel, and ionogels. (b) The right pattern is the magnified FTIR patterns from 1400 to 1900 cm^{-1}.

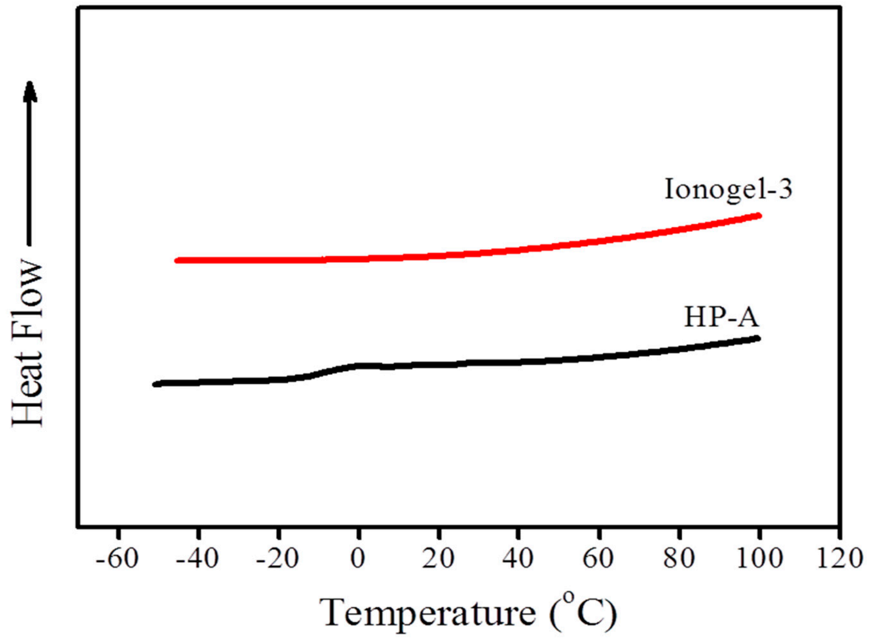

Figure S4 The melting DSC curves of HP-A and Ionogel-3 at a heating rate of 20 ºC/min.

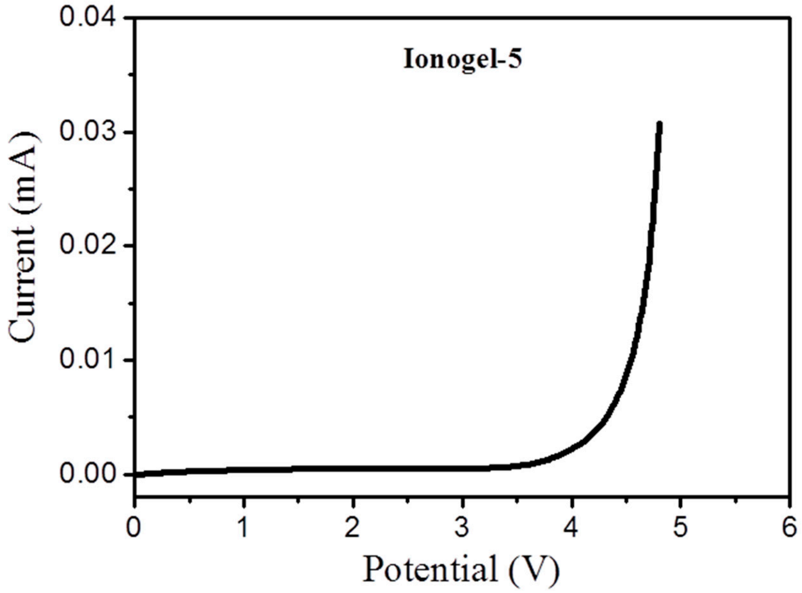

Figure S5 Linear sweep voltammograms of ionogel-5 at room temperature.
